# Type-3 Gaucher disease with bilateral necrosis of the neck of femur: a case report

**DOI:** 10.1186/1757-1626-2-9380

**Published:** 2009-12-22

**Authors:** Shobha Mohindroo

**Affiliations:** 1 Department of Pathology, Indira Gandhi Medical College, Shimla, Himachal Pradesh, India

## Abstract

**Introduction:**

Though Gauchers disease (G.D.), a lipid storage disease is most commonly encountered by the hematologist but we present a case of G.D. type-3 which is rare in Indian subcontinent.

**Case presentation:**

A 36-year-old Hindu, Indian male patient presented with bony pain, kyphosis, dragging sensation in the abdomen, dementia and ptosis of the left eyelid for one year. Clinical examination and history pointed to be a lipid storage disease. Radiological examination showed bilateral necrosis of the neck of femur. A final diagnosis of G.D. type-3 with bilateral necrosis of the femur neck was reported after examining the bone marrow smears. Unfortunately, we were unable to do the enzyme study as the patient was lost in the follow-up.

**Conclusion:**

G.D. type-3 with bilateral necrosis of femur neck is rare in our sub-continent. We are adding a new case to stress the importance of early recognition by clinical manifestation and finding of G.C. which is hallmark in the diagnosis of G.D.

## Introduction

G.D. an autosomal recessive spingolipid disorder resulting from the accumulation of glucocerebrosidase in the cells of macrophage-monocyte system as a result of a deficiency in lysosomal β-glycosidase [glucocerebrosidase] which is encoded by the gene on chromosome-1[1].

It was first described by Phillippe Gaucher in 1882, who thought that the peculiar large cells in the spleen were evidence of a primary neoplasm of spleen [2]. In 1924, Epstein first recognized the storage of glucocerebroside [3]. Brandy et al. delineated the metabolic defect, the deficiency of the lysosomal hydrolyse acid, β-glucosidase [4].

G.D. is the most common in Ashkenazi Jewish population in which the gene frequency is 0.034 with expected birth frequency 1:1000 [5]. It is also relatively common in the population isolate in Norrbottenia in Northern Sweden [6]. In the general population the frequency is rare, occurring in 1:50,000 to 1:1000,000 births as per Genetics Home reference Website [7].

## Case Presentation

**Figure 1:**
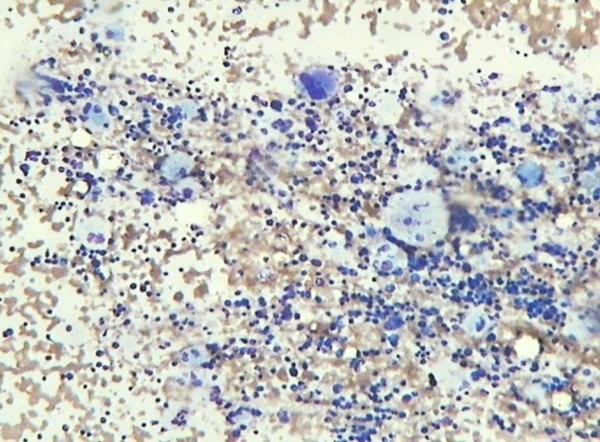
**Bone marrow smear showing numerous Gaucher cells-MGG ×10**.

**Figure 2:**
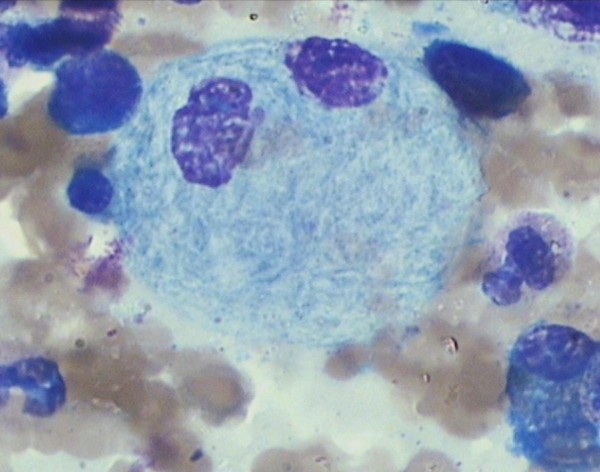
**Bone marrow smear showing Gaucher cell**. The cytoplasm has a fibrillary and striated appearance with nuclei small and eccentric in location MGG ×100

**Figure 3:**
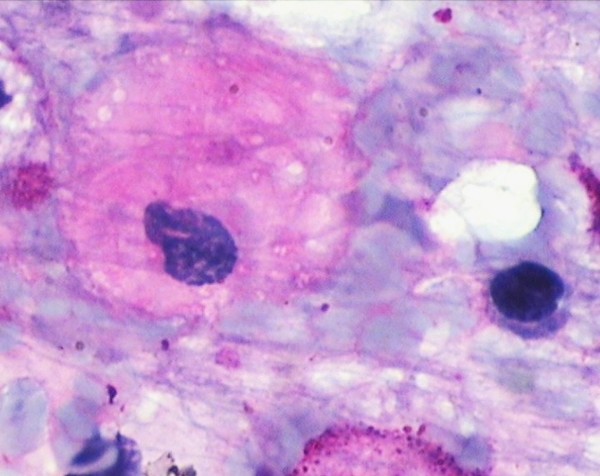
**Bone marrow smear showing Gaucher cells strongly positive with PAS reaction ×100**.

A 36-year-old Hindu, Indian male presented in the department of medicine I.G.M.C. Shimla with complaints of bony pain, limping, kyphosis, dragging sensation in the left abdomen and ptosis of the eyelids for about one year duration. There was a past history of epilepsy and seizure which is under control for the last twenty years. The general physical examination was normal. The haematological investigation found mild aneamia, total lecocyte count: 4,000/cumm, and platelets were 1.5 lakhs/cumm. Peripheral smear findings showed mild dimorphic anemia with eosinophilia. Bone marrow examination found particulate, cellular smears M:E: 3:1. Erythropoiesis was dimorphic. Megakaryocytes seen in adequate number and function. Myelogram showed myeloblast 03%, promyelocyte 01%, myelocyte 10%, metamyelocyte 20%, plasma cell 01%, polymorphs 47%, lymphocytes10%, eosinophills 08%. In addition, B.M. showed large number of large cells 30 to 100 microns in diameter having one to multiple, centrally or eccentrically placed nuclei with pale, blue-gray cytoplasm having characteristic fibrally and striated pattern [Fig 1 &2]. These cells stained strongly with P.A.S staining [Fig 3] and were Sudan Black negative. Keeping in view, the clinical presentation and the presence of Gaucher cells in the B.M., the G.C. infiltration into the B.M. with eosinophilia was suggested.

## Discussion

There are three clinical subtypes of G.D. that has been delineated by the absence or presence and progression of neurologic involvement. These are: Type 1 (the adult, non-neuronopathic form) occurring in children as well as adults with at least one mild mutation. Type -2 (the infantile or acute neuronopathic form) which is exceeding rare and characterized by rapid neurologic deterioration and early death. It does not occur predominantly in Jewish families.Type-3 (juvenile or Norrbotten form) is a well-defined subacute neuronopathic disorder with late onset of neurologic symptoms and has better prognosis than type-2.

The clinical course of G.D. depends on the clinical subtypes. Patients with type-1 disease have a broad spectrum of clinical expression in part due to combination of different mutant alleles. Onset of clinical manifestations are: splenomegaly, bone involvement, bone pain, pathological bone fracture, easy bruisability due to thrombocytopenia, chronic fatigue secondary to anaemia, hepatomegaly with or without elevated liver function tests. Occasionally, patients present with pulmonary involvement. Patients with a milder form of the disease present later in the life with haematological or skeletal complaints and/or have splenomegaly on routine examination. Children with massive splenomegaly are short statured due to increased metabolism by the enlarged spleen. Although the disease is progressive in the adults, it is compatible with long life.

Type-2 disease is rare and panethic in distribution and is characterised by a rapid neurodegenerative course with extensive visceral involvement and death within first two years of life. The disease is present in infancy with increased tone, strabismus, organomegaly, failure to thrive and laryngospasm.

Type-3 disease presents with neurological involvement in addition to organomegaly and bony manifestations.

G.D. should be considered in the differential diagnosis of patients with unexplained splenomegaly with an extended period of time or easy bruisability or bone pain. B.M. examination is the hallmark for the diagnosis of G.D however, all suspects should be confirmed by demonstrating deficient acid β-glucosidase activity in isolated leukocytes [8] or cultured fibroblasts [9] or by demonstrating the presence of known Gaucher mutations in the patients DNA.

Pseudo-Gaucher cells may be found in the marrow of some patients with CML, type II congenital dyserythropoietic anaemia, thalassemia, Hodgkin lymphoma, multiple myeloma and AIDS. These patients do not lack the capacity to catabolise glucocerbroside [10] but the great inflow of glucoside in to phagocytic cells exceeds their normal capacity to hydrolyze this glycolipid. Prenatal diagnosis of G.D. is established by examining cultured amniocentesis cells for their β-glucosidase activity [9] or by examining amniocentesis or chorionic villus DNA for mutations. A monoclonal gammopathy is present with chronic G.D. and may be associated with a marrow plasmocytosis [11]. The coincidence of multiple myeloma, plasmacytoid lymphoma and G.D. has also been reported [12].

Despite its rarity in India, we present this case to stress the importance of clinical presentation and B.M. finding in the diagnosis of G.D. This emphasis the relevance of an early detection of the disease.

## Abbreviations

G.D: Gauchers disease; G.C: Gauchers cells; I.G.M.C: Indira Gandhi Medical College; cumm: cubic millimeter; P.A.S: Periodic Acid Schiff; M.E.: Myeloid: Erythroid; B.M: Bone marrow; CML: Chronic myeloid leukemia; AIDS: Acquired immunodeficiency syndrome; MGG: May-Grunwald-Giemsa

## Competing interests

The author declares that they have no competing interests.

## Financial support

None

## Consent

An informed consent has been obtained from the patient and available with the Editor-in Chief.
